# Barcoding blood meals: New vertebrate-specific primer sets for assigning taxonomic identities to host DNA from mosquito blood meals

**DOI:** 10.1371/journal.pntd.0006767

**Published:** 2018-08-30

**Authors:** Lawrence E. Reeves, Jennifer L. Gillett-Kaufman, Akito Y. Kawahara, Phillip E. Kaufman

**Affiliations:** 1 Entomology and Nematology Department, Institute of Food and Agricultural Sciences, University of Florida, Gainesville, Florida, United States of America; 2 McGuire Center for Lepidoptera and Biodiversity, Florida Museum of Natural History, University of Florida, Gainesville, Florida, United States of America; National Institutes of Health, UNITED STATES

## Abstract

The transmission dynamics of mosquito-vectored pathogens are, in part, mediated by mosquito host-feeding patterns. These patterns are elucidated using blood meal analysis, a collection of serological and molecular techniques that determine the taxonomic identities of the host animals from which blood meals are derived. Modern blood meal analyses rely on polymerase chain reaction (PCR), DNA sequencing, and bioinformatic comparisons of blood meal DNA sequences to reference databases. Ideally, primers used in blood meal analysis PCRs amplify templates from a taxonomically diverse range of vertebrates, produce a short amplicon, and avoid co-amplification of non-target templates. Few primer sets that fit these requirements are available for the *cytochrome* c *oxidase subunit I* (*COI*) gene, the species identification marker with the highest taxonomic coverage in reference databases. Here, we present new primer sets designed to amplify fragments of the DNA barcoding region of the vertebrate *COI* gene, while avoiding co-amplification of mosquito templates, without multiplexed or nested PCR. Primers were validated using host vertebrate DNA templates from mosquito blood meals of known origin, representing all terrestrial vertebrate classes, and field-collected mosquito blood meals of unknown origin. We found that the primers were generally effective in amplifying vertebrate host, but not mosquito DNA templates. Applied to the sample of unknown mosquito blood meals, > 98% (60/61) of blood meals samples were reliably identified, demonstrating the feasibility of identifying mosquito hosts with the new primers. These primers are beneficial in that they can be used to amplify *COI* templates from a diverse range of vertebrate hosts using standard PCR, thereby streamlining the process of identifying the hosts of mosquitoes, and could be applied to next generation DNA sequencing and metabarcoding approaches.

## Author summary

Female mosquitoes take blood meals from diverse vertebrate hosts, including amphibians, birds, mammals, reptiles, and fishes. Mosquito species vary in their host-use patterns. Identifying which mosquitoes feed from which hosts is critical to understanding how mosquitoes transmit disease-causing pathogens between vertebrates. Host-use patterns are determined by blood meal analysis. In modern blood meal analysis, host DNA is extracted from a mosquito, amplified in a polymerase chain reaction (PCR), sequenced, and identified. We designed new primers that selectively amplify a fragment of the DNA barcoding gene, *cytochrome* c *oxidase subunit I* (*COI*), of vertebrates, while avoiding that of mosquitoes. We validated the new primers in experiments using previously identified mosquito blood meals and mosquito DNA, and demonstrated their efficacy in identifying unknown mosquito blood meals. We found that the primers are well-suited to mosquito blood meal analysis applications. They are universal across vertebrates, target the *COI* DNA barcoding gene, and are effective in standard amplification reactions.

## Introduction

Females of most mosquito species require a blood meal, taken from a host animal, in order to mature their eggs [1]. This requirement enables mosquitoes to vector disease-causing parasites and pathogens between their vertebrate host animals. Within an ecosystem, the host-use patterns of the mosquito community form a network that mediates the movement of mosquito-vectored parasites and pathogens between vertebrate hosts [2]. Determining the structure of these networks and the transmission dynamics of mosquito-vectored parasites and pathogens requires an understanding of the interactions between mosquito and vertebrate host communities. Such interactions define mosquito host-use patterns and are characterized through molecular or serological methods, collectively referred to as blood meal analysis, that determine the taxonomic origin of a mosquito blood meal [3, 4].

Current blood meal analysis approaches are largely PCR- and Sanger sequencing-based. In general, DNA is extracted from a blood meal, a taxonomically informative fragment of host DNA is amplified by PCR and sequenced, and the resulting sequence is compared against a DNA sequence reference database [5]. For the barcoding region of the vertebrate *cytochrome* c *oxidase subunit I gene* (*COI*), few primers are available that fit the needs of blood meal analysis applications. Those that are available are either specific to certain host classes [6, 7, 8], limiting the ability to detect the full range of hosts, or involve nested amplification reactions [9, 10] that require greater resource and effort investment. Similarly, primer sets, mostly vertebrate class-specific, are available for another DNA barcoding marker, *cyt* b [11–13]. However, taxonomic coverage for *cyt* b in publicly accessible databases is not as complete as *COI*.

Amplification of vertebrate DNA templates from blood meal-derived DNA extracts requires PCR primers deliberately designed to fit the unique needs of blood meal analysis. Primers must account for the presence of abundant non-target DNA (e.g., mosquito, symbiont, parasite [14]), host templates that may be derived from species of any vertebrate class [3], and the inherent degradation of target DNA templates due to digestion in the mosquito midgut. To address these factors, primer sets ideally (1) avoid co-amplification of non-vertebrate templates through vertebrate-mosquito priming site nucleotide mismatches, (2) amplify a universal range of vertebrate host taxa templates by annealing at sites that are conserved across vertebrate classes, and (3) produce a short amplicon. Balancing versatility across target taxa with avoidance of non-target co-amplification can be challenging, particularly for *COI* templates which may have limited conserved sites across target taxa [15]. Sequence variation between vertebrate taxa often requires the use of multiple degenerate sites on the primer sequence to achieve versatility. A degenerated primer sequence represents a population of distinct primer sequences that collectively cover the range of potential annealing site sequence combinations [16]. Increasing the number of degenerate bases in a primer sequence increases the number of unique primer sequences in the population. As a result, when primer sequences are highly degenerate (i.e., > 516-fold degeneracy), the likelihood of non-specific amplification increases [17, 18].

Based on these considerations, we designed four degenerate primer oligonucleotides that can be paired to amplify a 244, 395, or 664 bp fragment of the vertebrate *COI* gene. The primers were validated in two experiments. The first (Experiment 1) assessed primer versatility across vertebrate host classes and avoidance of mosquito template co-amplification using DNA extracted from a set of previously identified mosquito blood meals and unfed female and male mosquitoes. The second (Experiment 2) investigated the efficacy of the new primers in identifying a set of unknown mosquito blood meals.

## Methods

### Primer design

We designed two forward and two reverse primers intended to amplify templates on the barcoding region of the vertebrate *COI* gene [19] while excluding mosquito templates (Table 1). We downloaded *COI* sequences of 31 vertebrate host species belonging to the classes Amphibia, Aves, Mammalia, and Reptilia, and 12 mosquito species of the genera *Aedes*, *Anopheles*, *Culex*, *Culiseta* and *Uranotaenia* from the NCBI GenBank database [20] (S1 Table). The 43 reference sequences were aligned using the bioinformatics software Geneious 8.9.1 [21]. The alignment was used to identify 20–25 bp sequences that were well-conserved across vertebrate taxa but included nucleotide mismatch positions between mosquito and vertebrate taxa (vertebrate universal), or well-conserved across all taxa (universal). Three potential primer sequences containing primer-mosquito annealing site mismatches and one well-conserved potential primer sequence were identified from the alignment. Oligonucleotide primers were designed for these sites with sequence variation among vertebrate host taxa represented by degenerate bases [22]. To minimize the level of degeneracy of the primers, we allowed for limited primer-vertebrate annealing site mismatches, and designed the primers so that when such mismatches could not be avoided, they were positioned towards the 5' end of, or internally within the primer sequence, where they would be least likely to impede amplification [23].

**Table 1:** Primer sequences used in this study.

Primer label	Sequence	Specificity
Mod_RepCOI_F	5'- TNT TYT CMA C**Y**A A**C**C A**C**A AAG A -3'	Vertebrate universal
Mod_RepCOI_R	5'- TTC DGG RTG NCC RAA RAA TCA -3'	Universal
VertCOI_7194_F	5'- CGM ATR AAY AAY ATR AG**C** TT**C** TGA **Y** -3'	Vertebrate universal
VertCOI_7216_R	5'- CAR AA**G** CTY AT**G** TTR TTY ATD CG -3'	Vertebrate universal

Four primer sequences were designed to amplify a fragment of the barcoding region of the vertebrate *cytochrome* c *oxidase subunit I* (*COI*) gene based on a multiple alignment of 31 vertebrate species and 12 mosquito species. Nucleotide positions that are mismatched between all aligned mosquito reference sequences and the primer sequence are bolded. A nucleotide position that is mismatched between most, but not all, mosquito reference sequences is underlined.

Two primers, a vertebrate universal forward primer (Mod_RepCOI_F) and a universal reverse primer (Mod_RepCOI_R), were designed from an existing primer pair (RepCOI_F and RepCOI_R) that was originally created for DNA barcoding Madagascan reptiles [24] and has been used without modification in blood meal analysis targeting reptilian hosts [8]. The forward primer sequence and its position were modified to accommodate mosquito-vertebrate nucleotide mismatches toward the 3' end. Both forward and reverse primers were modified to improve versatility across vertebrate taxa. Two primers (VertCOI_7194_F and VertCOI_7216_R) were designed *de novo* at similar positions within the Mod_RepCOI amplicon. These four primers allow three primer combinations that produce an amplicon of 244, 395, or 664 bp (Table 2).

**Table 2:** Primer combinations used in this study.

Primer combination	Amplicon length (bp)
Mod_RepCOI_F + Mod_RepCOI_R	664
VertCOI_7194_F + Mod_RepCOI_R	395
Mod_RepCOI_F + VertCOI_7216_R	244

Three primer combinations were used to amplify a fragment of the barcoding region of the vertebrate *cytochrome* c *oxidase subunit I* (*COI*) gene. The length of the amplicon is dependent on the primer combination used.

### Experiment 1: Primer versatility and specificity

We tested the ability of the three primer combinations to amplify a diverse range of vertebrate host class templates (versatility), and to avoid the co-amplification of mosquito templates (specificity). To assess the versatility and specificity of each primer combination, we compared the DNA concentration of PCR products derived from several template categories: vertebrate host class templates (Amphibia, Aves, Mammalia, Reptilia), mosquito-only DNA templates, and no-DNA negative controls.

For the vertebrate host class categories, we used a set of 93 previously identified mosquito blood meal DNA extracts (S2 Table), each representing a unique vertebrate species (one blood meal per species) and together representing the vertebrate classes Amphibia (9 species), Aves (51 species), Mammalia (17 species), and Reptilia (16 species). This set of templates was selected to represent a wide range of vertebrate classes and species, from a larger set of blood meals field-collected in Florida, and identified using at least one of the primer combinations (Table 2) or another vertebrate-specific *COI* primer set [10, 24]. Sequencing trace files associated with the selected blood meal extracts contained unambiguous sequences, with no indication of the presence of DNA from multiple hosts (electropherogram double peaks) or degraded signal. All blood meals were fresh at the time of DNA preservation (blood meal scored as BF1 or BF2, as described in the *Field and laboratory protocols* section). Mosquito specimens from which the set of blood meal DNA templates were derived represented 17 species of the genera *Aedes*, *Anopheles*, *Culex*, *Culiseta*, *Psorophora*, *Uranotaenia*, and *Wyeomyia* (S2 Table). The mosquito-only DNA template category consisted of 14 DNA extracts derived from unfed female or male mosquitoes, each a unique species. DNA preservation and extraction protocols for these 14 mosquito-only extracts were identical to those used for blood meals extracts.

Each of the 93 vertebrate host templates, 14 mosquito-only DNA templates and four negative controls were used in three PCRs, each with one of the three primer combinations. Reactions were performed in 96-well PCR plates and all three PCRs per individual DNA extract were included on the same plate and thermocycler run. Amplification success was initially determined by ethidium bromide staining and gel electrophoresis of PCR products as described in the *Field and laboratory protocols* section. For each PCR product, the remaining volume was sent to the University of Florida, Interdisciplinary Center for Biotechnology Research (ICBR) for DNA quantification by Qubit fluorometer (Thermo Fisher Scientific, Waltham, MA). To account for differences in amplicon length, Qubit DNA concentration readings (ng/μl) were used to calculate the DNA concentration in nM and nanomolar concentrations were used in statistical comparisons. For each primer combination, mean DNA concentration of PCR products for the categories Amphibia, Aves, Mammalia, Reptilia, mosquito and negative controls were compared.

#### Statistics

Statistical analyses were performed in the software R Version 3.2.0 using the stats package [25]. Main and interactive effects of primer combination (three categories corresponding to the primer combinations listed in Table 2) and template (six categories: Mammalia, Aves, Reptilia, Amphibia, mosquito, negative control) on DNA concentration (nM) of PCR products were assessed using a fully crossed analysis of variance (ANOVA). Template was treated as a random effect and primer as a fixed effect. *Post hoc* pairwise comparisons were made using Tukey’s Honest Significant Differences (HSD) test at the 95% confidence level implemented in the R package *Agricolae* [26]. A one-way ANOVA was used to compare the mean DNA concentration (nM) of PCR products amplified by each primer combination, with mosquito templates and negative controls excluded.

### Experiment 2: Identification of unknown blood meals

To investigate the reliability and feasibility of our primer sets, we performed a small-scale test using field-collected blood meal specimens. We used a hierarchical approach to PCR amplify vertebrate host templates from the set of unknown blood meal DNA extracts collected at a field site in Alachua County, Florida. Initially, each DNA extract was used in one PCR with the Mod_RepCOI_F + VertCOI_7216_R primer combination. Ethidium bromide staining and gel electrophoresis were used to determine if amplification was successful. If amplification was unsuccessful, the DNA extract was used in a second PCR with the VertCOI_7194_F + Mod_RepCOI_R primer combination. If this reaction was not successful, the DNA extract was then used in a final PCR with the Mod_RepCOI_F + Mod_RepCOI_R primer combination. If amplification failed in all three reactions, no further steps were taken. If amplification was successful, the DNA extract was not used in subsequent reactions, and the PCR product was sequenced.

Products of all successful reactions were sent to Genewiz (South Plainfield, NJ) for DNA sequencing using Sanger sequencing on an ABI 3130 sequencer (Applied Biosystems, Foster City, CA). Resulting sequence chromatograms were examined and edited for quality in the bioinformatics software Geneious Version 8.9.1 [21]. Edited sequences were submitted to the BOLD v. 4 Identification Engine [27]. A species-level taxonomic identity was assigned to a sequence if it was ≥ 98% similar [28] to a sequence referenced in the BOLD database or to an independently obtained reference sequence. In several cases, blood meal *COI* sequences submitted to BOLD did not meet this criterion, but were suspected to either represent a species not yet referenced in the BOLD database (i.e., *Sylvilagus palustris*; marsh rabbit) or a species with unusually high intraspecific *COI* sequence divergence (i.e., *Anolis carolinensis*; green anole). In Florida, there are several distinct lineages of *A*. *carolinensis* that correspond with ancient island refugia [29, 30]. None of the reference sequences currently in the BOLD database that include locality information represent the *A*. *carolinensis* lineage (central Florida) that occurs in the study region. For *A*. *carolinensis* and *S*. *palustris*, we independently obtained reference sequences for comparison against blood meal sequences. Extracted DNA from tissue of morphologically identified specimens collected in central Florida were provided for *A*. *carolinensis* (University of Florida, Florida Museum of Natural History, Division of Herpetology; accession numbers 170869 and 170871) and *S*. *palustris* (University of Florida, Florida Medical Entomology Laboratory, collected by Nathan Burkett-Cadena; IACUC protocol number 201408377). From these DNA extracts, we generated reference sequences using the Mod_RepCOI_F + Mod_RepCOI_R primer combination (664 bp) in PCR and sequencing, as described in the *Field and laboratory protocols* section. These reference sequences were aligned to blood meal sequences that did not meet the ≥ 98% similarity criterion using the NCBI Basic Local Alignment Search Tool (BLAST) for two sequences. If similarity was ≥ 98%, the corresponding taxonomic identity was assigned to the sequence.

### Field and laboratory protocols

#### Mosquito collection

All mosquitoes used in Experiment 1 were collected from field sites in Florida from 2015–2017 (S2 Table) with a battery-powered, handheld aspirator, made from a modified 18 v Dustbuster vacuum (BDH1800S, Black and Decker), from either natural (e.g., vegetation, tree trunks, roots of fallen trees) or artificial resting sites. Artificial resting sites were constructed after Burkett-Cadena [31]. Specimens collected from Everglades National Park, Florida, USA, were included in Experiment 1, and were collected under permit numbers EVER-2015-SCI-0054 and EVER-2017-SCI-0011. Permits were not needed for mosquito specimens collected elsewhere.

For Experiment 2, a set of blood fed mosquitoes was collected at River Styx, a bottomland swamp in southern Alachua County, Florida, USA (29.51891°, -82.21701°) on 28 April 2017. The same battery-powered aspirator described above was used to collect resting mosquitoes from natural resting sites and the forest floor. Sampled mosquitoes were promptly transported 25 km to the Entomology and Nematology Department, University of Florida, Gainesville, Florida, USA, where they were killed and examined under a stereoscope. Blood meal DNA was preserved and extracted from any mosquito that contained a visible trace of a blood meal, resulting in a set of unknown blood meal DNA extracts.

#### Blood meal preservation and DNA extraction

The protocols used to preserve and extract blood meal DNA were identical for Experiments 1 and 2. Upon collection, mosquitoes were killed by exposure to ethyl acetate-soaked plaster in a 473 mL glass jar for approximately ten minutes, and examined under a stereoscope. Blood fed females were separated and identified to species using morphological keys [32]. Host DNA from each blood meal was preserved on Whatman four-sample Flinders Technology Associates (FTA) cards (Sigma-Aldrich Corp., St. Louis, MO) [33]. Specimens were transferred individually to the sampling area of a FTA card using forceps. Pressure was applied to the abdomen with a sterile pipette tip until the blood meal was released onto the card. The mosquito was discarded, and the pipette tip was used to spread the blood meal across the surface of the FTA card until all viscous droplets were absorbed. Each blood meal was given a unique identifying number and the FTA cards were stored in zipper-sealed plastic bags at room temperature inside a laboratory bench drawer until DNA extraction.

**Fig 1:**
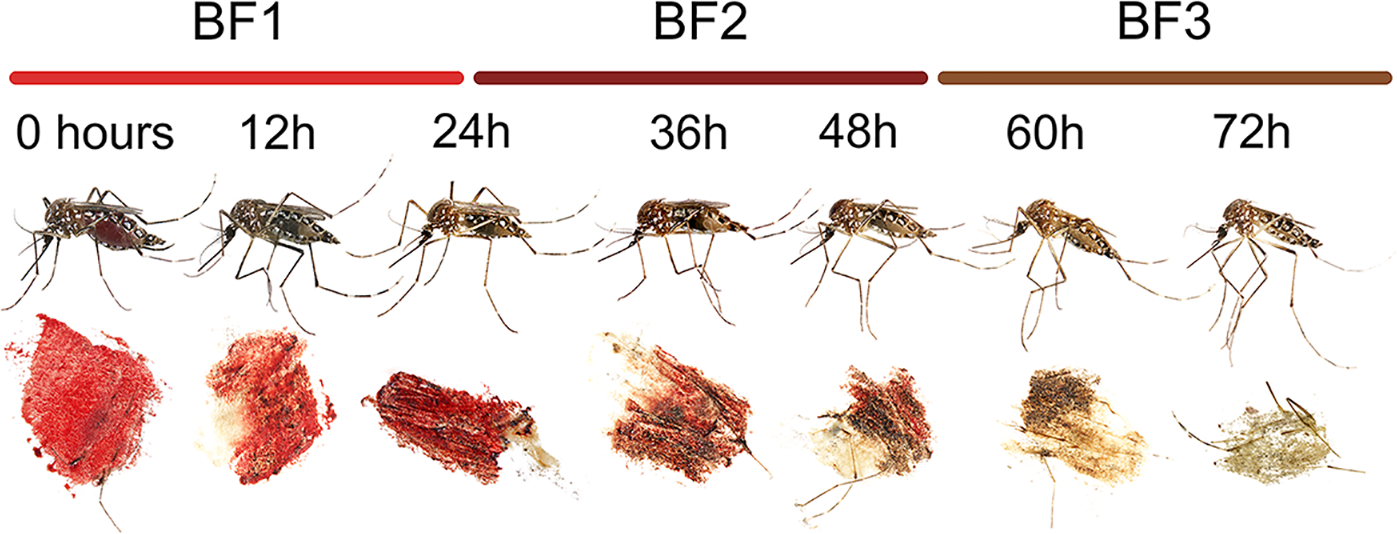
Blood meal scoring scheme, modified from Detinova [34], to estimate the extent of digestion in blood fed mosquitoes. Fresh blood meals were categorized as BF1, and were characterized by a large size, bright red color, and the absence of developing ovaries. Blood meals categorized as BF2 were intermediate in digestion extent, and had darker red blood meals with developing ovaries not taking up more than 50% of the abdomen. Blood meals categorized as BF3 were in later stages of digestion, brown in color, and had ovaries that took up more than 50% of the abdomen. This is illustrated with blood fed *Aedes aegypti* killed between 0 and 72 h post-feeding.

The extent of digestion of each blood meal was visually estimated (Fig 1) using an approach modified from Detinova [34] before and as the blood meal was preserved. Blood meals were scored (BF1-3) based on the color and size of the blood meal and the state of developing ovaries as indicated in Fig 1.

The HotSHOT method [35] was used to extract DNA from the FTA card-preserved blood meal specimens. For each blood meal, a hole punch was used to remove two circular, 1 mm diameter sections of the sampling area. The two cut-out punches were transferred to the same well of a 96-well PCR plate with flame-sterilized forceps. After each well contained two punches, 50 μL lysis solution (25 mM NaOH and 0.2 mM EDTA) was added to each. The plate was then incubated in a BioRad DNA Engine thermocycler at 95°C for 30 min followed by 4°C for five min. The plate was removed and 50 μL of neutralization solution (40 mM Tris-HCl) was added to each well. The plate was vortexed for ~10 s and stored at -20°C until PCR amplification.

#### PCR amplification

In Experiment 1 and 2, all three primer combinations (Table 2) were used to amplify vertebrate *COI* templates in separate reactions. With the exception of the primer combination, identical thermocycling conditions and PCR reagents, and their concentrations, were used in all reactions. Each PCR was performed in a total volume of 20 μL that consisted of 10 μL of 2.0X Apex Taq RED Master Mix (Genesee Scientific Corp., San Diego, CA), 0.75 μL of forward primer (10 μM), 0.75 μL of reverse primer (10 μM), 7.5 μL sterile, double-distilled water and 1 μL of extracted DNA. Thermocycling conditions were 94°C for 3 min, followed by 40 cycles of 94°C for 40 s, 48.5°C for 30 s, and 72°C for 60 s, and a final extension step of 72°C for 7 min. Negative controls in which sterile double-distilled water replaced extracted DNA were included in all reactions to monitor for contamination. For each PCR product, 7 μL were loaded into a well in an ethidium bromide-stained 1.5% agarose gel, and electrophoresed for approximately 30 min. Reaction products were subsequently visualized under ultra-violet light to determine amplification success. A 50 bp DNA ladder (Invitrogen, Waltham, MA) loaded alongside PCR products was used to determine if amplification was successful based on the presence of a band at the expected fragment length. We categorized bands on the gel as strong, weak, or no amplification based on the presence and intensity of bands at the expected fragment size.

## Results

### Experiment 1: Primer versatility and specificity

Gel electrophoresis indicated that amplification success for all primer combinations was generally high for blood meal templates across the range of vertebrate host classes, and poor for templates that contained only mosquito DNA (Table 3). However, no primer combination successfully amplified all blood meal templates, and for each combination, there were a small number of dim bands or failed amplifications. No PCR products were detected in the no-DNA negative controls. Amplification failed for the majority of reactions that used DNA extracted from unfed female or male mosquitoes. Faint bands at the expected amplicon size were visible on the gel following electrophoresis for DNA templates derived from *Aedes triseriatus* (all three primer combinations), *Uranotaenia sapphirina* (two primer combinations), and *Uranotaenia lowii* (one primer combination), indicating amplification of a low concentration PCR product. Otherwise, amplification of mosquito templates was undetected by gel electrophoresis.

**Table 3:** Amplification success of vertebrate host mosquito only DNA templates and no DNA controls.

Template	Amplification	Mod_RepCOI_F + Mod_RepCOI_R	VertCOI_7194_F + Mod_RepCOI_R	Mod_RepCOI_F + VertCOI_7216_R
**Amphibia** **(n = 9)**	**Strong**	8	8	6
	**Weak**	0	1	2
	**None**	1	0	1
**Aves** **(n = 51)**	**Strong**	47	46	50
	**Weak**	3	3	0
	**None**	1	2	1
**Mammalia** **(n = 17)**	**Strong**	15	16	11
	**Weak**	2	0	4
	**None**	0	1	2
**Reptilia** **(n = 16)**	**Strong**	14	15	16
	**Weak**	2	1	0
	**None**	0	0	0
**Mosquito** **(n = 14)**	**Strong**	0	0	0
	**Weak**	1	2	3
	**None**	13	12	11
**No DNA** **(n = 4)**	**Strong**	0	0	0
	**Weak**	0	0	0
	**None**	4	4	4

Amplification success was assessed by gel electrophoresis of ethidium bromide-stained PCR products. DNA templates for the vertebrate host classes Amphibia (9 species), Aves (51 species), Mammalia (17 species), and Reptilia (16 species), and mosquitoes (14 species) each represent a unique species. All DNA templates for vertebrate host classes were derived from mosquito blood meals preserved at the early stages of digestion (BF1-2), and because abundant non-target (i.e., mosquito) DNA was present, host DNA concentrations could not be standardized prior to PCR. Each DNA template was amplified once by each primer combination.

The results of Qubit fluorometer DNA concentration quantification of PCR products reflected those of gel electrophoresis. The majority of PCR products derived from blood meal templates contained DNA concentrations that were sufficient for Sanger sequencing (>45 nM), although there were some exceptions (S3 Table**)**. Qubit DNA concentration quantification, like gel electrophoresis, indicated that there were a small number of failed reactions and low concentration PCR products among blood meal templates. Primer combination Mod_RepCOI_F + VertCOI_7216_R was the most effective at amplifying high concentration PCR products, and the DNA concentration of PCR products was >45 nM for all 93 vertebrate host templates tested (S4 Table). However, an amplicon was not visible by gel electrophoresis in four cases. Comparatively, primer combinations VertCOI_7194_F + Mod_RepCOI_R and Mod_RepCOI_F + Mod_RepCOI_R each failed to produce >45 nM PCR products for nine and four vertebrate templates, respectively. DNA concentration of blood meal PCR products varied substantially across vertebrate host classes. The DNA concentration of PCR products from mosquito-only templates and no DNA negative controls was generally low. All negative controls resulted in products that contained a DNA concentration < 45 nM, likely representing primer interactions. In some cases (e.g., *Ae*. *triseriatus*, *Ur*. *lowii*, *Ur*. *sapphirina*), PCR products from mosquito-only templates had DNA concentrations > 45 nM (S4 Table), but these concentrations were not consistently detectable by gel electrophoresis.

**Fig 2:**
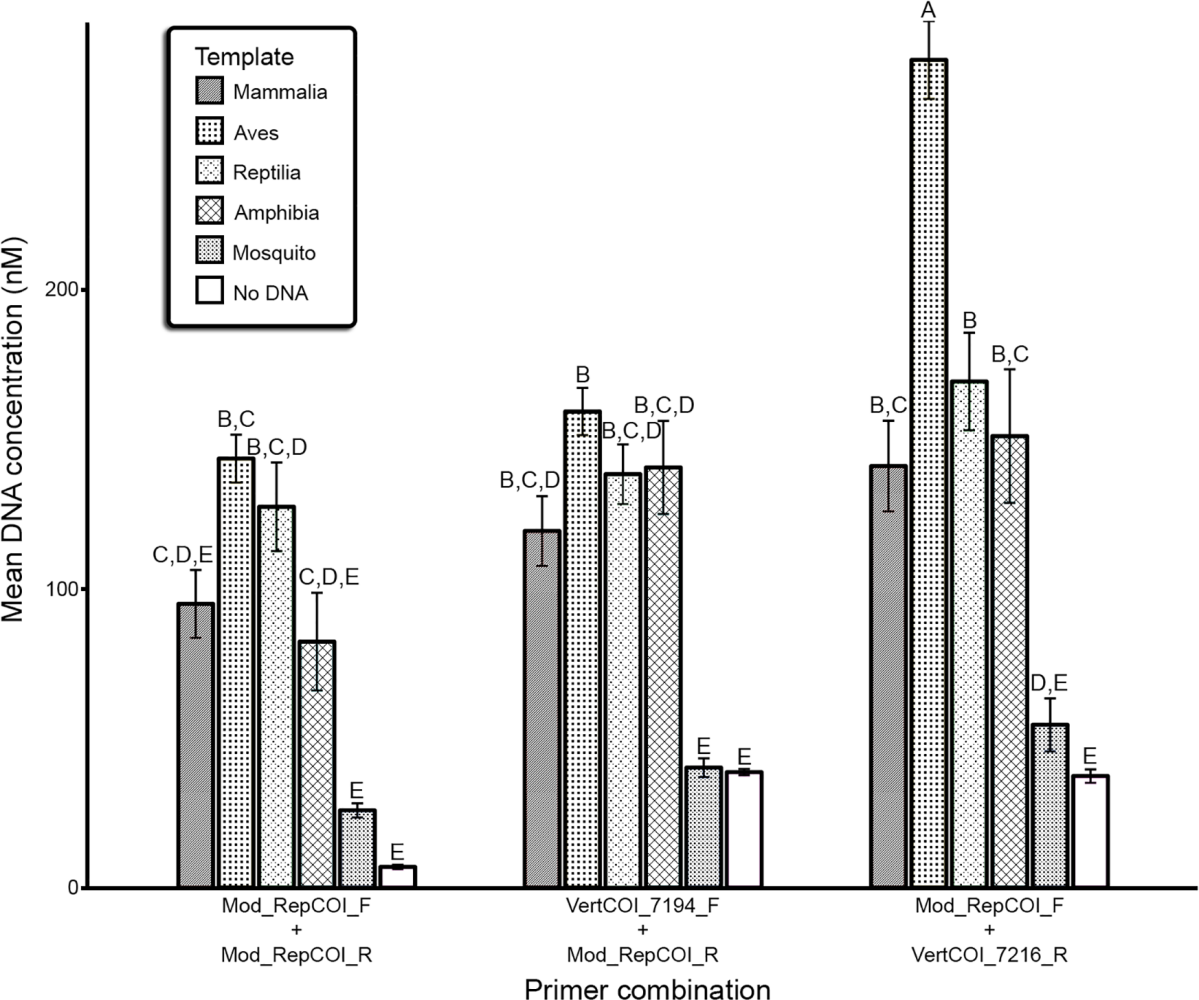
Mean (±SEM) DNA concentration (nM) of PCR products, measured by Qubit fluorometer, of the six template categories (Mammalia, Aves, Reptilia, Amphibia, Mosquito, negative control [No DNA]) by three primer combinations (Mod_RepCOI_F + Mod_RepCOI_R, VertCOI_7194_F + Mod_RepCOI_R, Mod_RepCOI_F + VertCOI_7216_R). Tukey’s HSD test detected pairwise differences between mean DNA concentration for each primer combination and host class. Significant differences between groups are indicated by letters. Mean DNA concentration of groups that have the same letter are not significantly different from each other, while mean DNA concentration of groups that do not share a common letter are significantly different. Comparisons were considered significant if *P* < 0.05. Error bars represent standard error of the mean.

Amplification success, as measured by the DNA concentration of PCR products, was significantly affected by primer combination, template and their interaction term (Table 4). Tukey’s HSD tests were used *post hoc* to compare the categories of template: the four host classes (Mammalia, Aves, Reptilia, Amphibia), mosquito, and the negative controls, among primer combinations. For all primer combinations, the DNA concentration of mosquito template and negative control PCR products were not significantly different from each other. Both mosquito template and negative control products were significantly different from all vertebrate categories of the template variable for all primer combinations, with the exception of the Mammalia and Amphibia categories amplified with the Mod_RepCOI_F + Mod_RepCOI_R primer combination (Fig 2). These results suggest that in general, the primer combinations are effective in parsing host templates from mosquito templates.

**Table 4:** Results of fully crossed analysis of variance (ANOVA).

Source of Variation	Degrees of freedom	Sum of Squares	Mean Square	*F*	*P*
Primer	2	434,647	217,323	61.03	<0.001
Template	5	1,031,409	206,282	57.93	<0.001
Primer*Template	10	174,253	17,425	4.89	<0.001
Residuals	315	1,121,671	3,561		

The effect of primer combination (Mod_RepCOI_F + Mod_RepCOI_R, VertCOI_7194_F + Mod_RepCOI_R, Mod_RepCOI_F + VertCOI_7216_R), template (Amphibia, Aves, Mammalia, Reptilia, mosquito, negative [no DNA] controls) and their interaction on the DNA concentration (nM) of PCR products were tested.

To better interpret differences between the amplification success of vertebrate templates amplified by each primer combination, we compared the mean DNA concentration of PCR products of the primer combinations in a one-way ANOVA with the mosquito template and negative control categories of the template variable omitted. Primer combination had a significant effect on amplification success (*F*_2,267_ = 234,858, *P* = <0.001). Post hoc Tukey’s HSD tests (*P* < 0.05) indicated that, with mosquito template and negative control categories omitted, mean DNA concentration was significantly higher for Mod_RepCOI_F + VertCOI_7216_R. No differences were detected between Mod_RepCOI_F + Mod_RepCOI_R and VertCOI_7194_F + Mod_RepCOI_R.

### Experiment 2: Identification of unknown blood meals

Altogether, 61 blood-fed and two unfed female mosquitoes were collected at River Styx, Alachua County, Florida, USA on 28 April 2017 (S4 Table), representing six species (Table 5). In the first PCR, using the primer combination Mod_RepCOI_F + VertCOI_7216_R, amplification of vertebrate *COI* templates was successful for 59 of 61 (96.7%) blood meal DNA extracts. Amplification was not detected by gel electrophoresis of ethidium bromide-stained PCR products for the two negative control unfed females. Sanger sequencing reactions for successful PCRs resulted in high quality, unambiguous *COI* sequences that were each reliably matched to a vertebrate host species. In all cases, no indications of multiple host feedings were apparent in sequencing trace files. With the exception of mosquitoes that had fed on *Anolis* lizards and *Sylvilagus* rabbits, all sequences met the ≥ 98% similarity criterion for reliable identification when submitted to BOLD. Twenty-two *Cx*. *territans* (69% of individuals of this species) and five *Cs*. *melanura* (83%) had taken blood meals from hosts suspected to be *A*. *carolinensis* based on BOLD query results. Sequences from these individuals were close (93 to 96% similarity) matches to *A*. *carolinensis* and *Anolis porcatus* (Cuban green anole) BOLD reference sequences, but did not meet the ≥ 98% similarity criterion to attribute species-level identifications to the samples. Of the two species, only *A*. *carolinensis* is known from northern/central Florida, while *A*. *porcatus* is potentially established in extreme southern Florida [36]. Two-sequence BLAST alignments to independently obtained *A*. *carolinensis* reference sequences resulted in ≥ 98% similarity in all cases. Host *COI* sequences from two *Ae*. *infirmatus* blood meals were ~88% similar to BOLD-referenced *Sylvilagus audubonii* (desert cottontail) sequences. Because two *Sylvilagus* species, *Sylvilagus floridanus* (eastern cottontail) and *S*. *palustris* are known from Florida, and the BOLD database currently only includes reference sequences for *S*. *floridanus*, we suspected that these blood meal-derived sequences represented *S*. *palustris*. Alignment of these sequences to independently obtained *S*. *palustris* reference sequences resulted in ≥ 98% similarity in both cases.

**Table 5:** Results of blood meal analysis performed using newly designed *cytochrome* c *subunit I* primers.

Host species	*Aedes infirmatus*	*Anopheles crucians*	*Coquillettidia perturbans*	*Culex erraticus*	*Culex territans*	*Culiseta melanura*
*Odocoileus virginianus*	10	1	4	0	0	0
*Dasypus novemcinctus*	2	0	0	0	0	0
*Sylvilagus palustris*	2	0	0	0	0	0
*Cardinalis cardinalis*	0	0	0	0	0	1
*Butorides virescens*	0	0	0	1	0	0
*Nycticorax nycticorax*	0	0	0	2	0	0
*Hyla cinerea*	0	0	0	0	6	0
*Hyla femoralis*	0	0	0	0	3	0
*Hyla squirella*	0	0	0	0	1	0
*Anolis carolinensis*	0	0	0	0	22	5
No amplification	0	0	1	0	0	0
Total	14	1	5	3	32	6

Mosquitoes were collected at River Styx, Alachua Co., Florida, USA, on 28 April 2017. Values indicate the number of blood meals derived from a particular host species. All blood meals were identified using the Mod_RepCOI_F + VertCOI_7216_R primer combination, except one *Culex territans* blood meal that was identified with VertCOI_7194_F + Mod_RepCOI_R.

Extracted DNA from two mosquito blood meals (one *Cx*. *territans* and one *Cq*. *perturbans*) did not amplify in the initial PCR using the primer combination Mod_RepCOI_F + VertCOI_7216_R. Both DNA extracts were used in a second reaction using the primer combination VertCOI_7194_F + Mod_RepCOI_R. Extracted DNA from the *Cx*. *territans* blood meal amplified and was identified as *A*. *carolinensis*. The amplification failure in the initial reaction for this blood meal, scored BF2, may have been the result of a pipetting or protocol error, as its subsequent identification as *A*. *carolinensis* suggests that host DNA degradation or primer-annealing site mismatch were not the cause. A third PCR using the Mod_RepCOI_F + Mod_RepCOI_R combination failed to produce amplification of the remaining *Cq*. *perturbans* DNA extract. The *Cq*. *perturbans* blood meal may have failed because of an advanced stage of digestion (BF3), which correlates with degradation of host DNA templates.

## Discussion

The identification of generally universal *COI* primers for mosquito blood meal analysis that can be used in standard PCRs improves the ability to detect the full range of potential vertebrate hosts, reduces the resources and time required to make identifications, and streamlines the process of identifying the hosts of mosquitoes. We conclude that the new primers presented here are generally effective in amplifying a PCR product from a range of vertebrate host class DNA templates, but not mosquito DNA templates, and demonstrate their efficacy in identifying the host vertebrate species of unknown mosquito blood meals. These primers provide another option to the currently available *COI* primers for mosquito blood meal analysis, many of which are vertebrate class-specific, or otherwise require nested PCRs or primer cocktails [6–10]. Principal advantages of these new primers are that they amplify a fragment of the *COI* gene, are compatible with standard amplification reactions, vary in size and include a set producing a small (244 bp) amplicon, and amplify templates across a range of vertebrate classes. Concomitantly, these primers have limitations and could be improved. Importantly, no individual primer combination produced PCR products that could be detected by gel electrophoresis for the full range of tested vertebrate host species. This suggests that when applied to field-collected samples of unknown origin, none of the primer combinations alone should be expected to independently assure the amplification of a PCR product, necessitating a hierarchical approach.

One of the advantages of the *COI* gene in species identification is its rapid rate of evolution, and the resultant ability to distinguish between even recently diverged species [28]. Simultaneously, rapid rates of evolution complicate primer design for blood meal analysis by making it difficult to identify suitable priming sites consisting of sequences that are conserved across vertebrate taxa [15], but mismatched with mosquito templates. In cases where primer-template mismatches prevent the extension of host templates, a hierarchical approach can solve this issue through the use of varied priming sites in secondary or tertiary reactions that are expected to improve the likelihood that the template can ultimately be amplified. Using the primer combinations presented here, there are several factors that should be considered when designing a hierarchical PCR strategy (Table 6), including amplicon length and versatility differences between primer combinations.

**Table 6:** Summary of results and factors for consideration in designing a hierarchical approach to PCR using three combinations of new vertebrate-specific *cytochrome* c *oxidase subunit I* primers.

Primer combination	Amplicon size (bp)	Considerations for use
Mod_RepCOI_F + VertCOI_7216_R	244	Short amplicon ideal for degraded DNA templates under the assumption that short DNA fragments are present in a greater abundance than long fragments in well-digested mosquito blood meals. However, shorter fragments may not contain sufficient sequence variation to resolve differences between closely related taxa, and are more susceptible to NUMT co-amplification. In these cases, a subsequent PCR with another primer combination, targeting a longer fragment will be necessary. Yields generally high DNA concentration products across wide range of host taxa, especially for avian templates. Recommended for the initial reaction of a hierarchical approach because amplicon size is small, and amplicon yields are generally high.
VertCOI_7194_F + Mod_RepCOI_R	395	Generally good DNA yields relatively consistent across vertebrate classes, but may fail with a small proportion of vertebrate taxa. Well suited for secondary reactions. Relatively short amplicon useful for degraded templates, and unlikely to encounter problems in differentiating between closely related host species. Recommended for the secondary reaction of a hierarchical approach because amplicon size is relatively small, and primer annealing site differs from Mod_RepCOI_F + VertCOI_7216_R combination.
Mod_RepCOI_F + Mod_RepCOI_R	664	DNA concentration of PCR products highest for avian and reptilian hosts. For amphibian and mammalian hosts, DNA concentration may be lower, but in general sufficient for Sanger sequencing. Suitable for secondary or tertiary reactions, or subsequent reactions to differentiate between closely related host species or resolve potential NUMT co-amplification issues. Longer amplicon poorly suited to amplify degraded host DNA templates (i.e., well-digested blood meals), as longer DNA templates are expected to persist in the mosquito gut for a shorter duration than short templates. Best used to resolve instances of ambiguous results produced by the other primer combinations.

Ideally, field-collected mosquito blood meals are fresh when collected and host DNA templates have undergone minimal digestive degradation. After a mosquito takes a blood meal, host DNA gradually degrades until becoming undetectable by PCR 30–72 h post-feeding [37–40]. As DNA degrades, strand breaks accumulate on template DNA molecules over time. As a result, shorter fragments persist for a longer period of time than long fragments, corresponding with an inverse relationship between amplification success and the size of the amplified template [41]. Under the assumption that shorter DNA fragments likewise persist for a greater duration in the mosquito midgut than longer fragments, we designed two primer combinations, VertCOI_7194_F + Mod_RepCOI_R and Mod_RepCOI_F + VertCOI_7216_R, that amplify relatively short fragments (395 and 244 bp, respectively) of template DNA and are expected to be more effective than primer sets that amplify longer fragments when blood meals are well-digested. Concomitantly, these shorter amplicons contain less taxonomic information and sequence variation than longer amplicons, potentially making it difficult to distinguish between closely related species [42]. Sequences of the *COI* DNA barcode region are expected to produce inconclusive identifications (98–100% identity to more than one species) for approximately 5% of taxa when the amplicon size is 250 bp [42]. In cases where the resulting sequences match multiple, closely-related sympatric host vertebrates, a subsequent PCR using a primer set producing a longer amplicon could be used to resolve ambiguity. Another issue in using shorter mitochondrial sequences for species identification is the potential for accidental amplification of nuclear mitochondrial pseudogenes (NUMTs; fragments of mitochondrial DNA transposed to the nuclear genome) [43]. Nuclear mitochondrial pseudogenes are most often <600 bp in length, usually <200 bp [44], and may be co-amplified. Co-amplification of a NUMT is expected to result in a trace file with ambiguous nucleotide peaks, which may lead to the inability to identify a sequence. Such trace files should be carefully examined and interpreted, not only to recognize NUMTs, but also blood meals of mixed origin that can produce similar ambiguous peaks [10]. Issues related to NUMTs are likely to be resolved by re-amplifying and sequencing a sample with a primer combination that produces a longer amplicon.

Experiment 2 tested the effectiveness of a hierarchical approach to blood meal identification using the newly designed primers on a small sample of field-collected mosquitoes. Although this sample was relatively limited in size and the number of mosquito and host species it represented, we were able to identify > 98% of the collected blood meals (60 of 61 blood meals). Contemporary literature records of mosquito blood meal identification success vary, but host identification success of ~50–80% of blood fed specimens are common [7, 45–48]. While we cannot expect that the high rate of amplification and host identification we observed in our sample will necessarily translate to larger samples or samples representing greater host diversity, these results support the validation of these primer combinations as an effective means of identifying mosquito blood meals.

In general, the mosquito-vertebrate host associations we determined in Experiment 2 reflect the known vertebrate host class associations of the collected mosquito species in North America [49–52]. However, our sample of *Cs*. *melanura* (n = 6), a typically ornithophilic mosquito and the primary vector of the *Eastern equine encephalitis virus*, a medically important *Alphavirus*, fed predominantly on the lizard *A*. *carolinensis*, and only one blood meal was derived from a bird. Previous research indicates that birds, particularly passerines, are dominant hosts for *Cs*. *melanura*. Much of the ecological research on *Cs*. *melanura* has taken place in the northern United States, beyond the range of *A*. *carolinensis* and other abundant lizard species, or has used molecular methods specific only to avian and mammalian hosts [53–55]. Our small sample size for *Cs*. *melanura* inhibits the ability to draw conclusions regarding general host-use patterns. However, this result reflects recent findings on *Cs*. *melanura* host-use patterns in Florida suggesting that *Anolis* lizards are important hosts for this mosquito in the state [56], and highlights the importance of using blood meal analyses that are compatible with the full range of potential mosquito host animal classes so that unexpected host taxa are not missed.

As molecular technologies advance and the costs of DNA sequencing decrease, next generation DNA sequencing is likely to be increasingly applied to examinations of vector blood meals. Technologies such as Illumina and 454 pyrosequencing have advantages over Sanger sequencing approaches to mosquito blood meal analysis [57], and make feasible a metabarcoding or community sequencing approach to identifying pooled mosquito blood meals. Under such an approach, DNA extracted from mosquito blood meals could be pooled and sequenced in parallel, enabling the identification of large numbers of blood meals simultaneously and affordably, albeit without the ability to link a particular host species to an individual mosquito. Primer sets that target only a range of the potential vertebrate host species or certain genes (e.g., *cyt* b, *16S ribosomal RNA*) may not lead to accurate characterizations of mosquito host-use patterns because feedings on non-target or unanticipated hosts could be missed, or host species for which reference sequences do not yet exist would not be identified, respectively. Similarly, primer sets that require nested amplification reactions can produce biased results when used to sequence communities [58]. The primer sets presented here may be useful in community sequencing approaches to blood meal analysis, as they are generally vertebrate-universal and compatible with standard PCR. However, variation in the efficiency of amplification between host taxa may pose an issue, and further research is needed to investigate the suitability of these, or other primers to next generation and community sequencing blood meal analysis approaches. Future research should also consider the possibility that some mosquitoes are specialists of non-vertebrate hosts [59], and strive to develop blood meal analysis methods that can detect the full range of animals that may be fed upon by mosquitoes.

Mosquito blood meal analysis provides insight on the host-use patterns of mosquito communities, and by extension, the ecology and epidemiology of mosquito-vectored pathogens. The *COI* primers presented here amplify *COI* templates of a universal range of terrestrial vertebrate classes while avoiding co-amplification of mosquito templates, and provide an alternative to the currently available primer sets that target only particular vertebrate classes, or require nested or multiplexed PCR. These primers streamline the process of determining the hosts of mosquitoes through Sanger sequencing, and are candidates for the development of next generation sequencing or metabarcoding-based approaches to blood meal analysis.

## Supporting information

S1 Table National Center for Biotechnology Information (NCBI) GenBank accession numbers for mosquito and vertebrate sequences that were aligned and used to identify and design potential vertebrate-specific primers. (DOCX)

S2 Table Taxonomic details and collection information for known blood meal samples used to test the effectiveness and versatility of newly designed primers. Mosquito-only DNA samples used to ensure that mosquito templates were not amplified are listed under Culicidae.
(DOCX)

S3 Table DNA concentration (nM) of PCR products amplified using three primer combinations and templates from various vertebrate host and mosquito species, and negative controls. Concentrations were measured by Qubit fluorometer and converted from ng/μl to nM to account for differences in amplicon length between primer combinations.
(DOCX)

S4 Table Mosquitoes collected at River Styx, Alachua Co., FL, USA, digestion extent and vertebrate host identification. (DOCX)

## References

[1] Downes JA. The feeding habits of biting flies and their significance in classification. Annu. Rev. Entomol. 1958;3: 249–266.

[2] Chaves LF, Harrington LC, Keogh CL, Nguyen AM, Kitron UD. Blood feeding patterns of mosquitoes: Random or structured? Front Zool. 2010;7: 3 10.1186/1742-9994-7-3 20205866 PMC2826349

[3] Tempelis CH. Host-feeding patterns of mosquitoes, with a review of advances in analysis of blood meals by serology. J Med Entomol. 1975;11: 635–653. 235647 10.1093/jmedent/11.6.635

[4] Washino RK, Templis CH. Mosquito host bloodmeal identification. Annu Rev Entomol. 1983;28: 179–201. 6131641 10.1146/annurev.en.28.010183.001143

[5] Kent RJ. Molecular methods for arthropod bloodmeal identification and applications to ecological and vector-borne disease studies. Mol Ecol Resour. 2009;9: 4–18. 10.1111/j.1755-0998.2008.02469.x 21564560

[6] Ivanova NV, Dewaard R, Hebert PDN. An inexpensive, automation-friendly protocol for recovering high-quality DNA. Mol Ecol Notes. 2006;6: 998–1002.

[7] Navia-Gine WG, Loaiza JR, Miller MJ. Mosquito-host interactions during and after an outbreak of equine viral encephalitis in eastern Panama. PLoS ONE. 2013;12: e81788. 10.1371/journal.pone.0081788 PMC3858258 24339965

[8] Reeves LE, Krysko KL, Avery ML, Gillett-Kaufman JL, Kawahara AY, Connelly CR, Kaufman PE. Interactions between the invasive Burmese python, *Python bivittatus* Kuhl, and the local mosquito community in Florida, USA. PLoS ONE. 2018;14: e0190633. 10.1371/journal.pone.0190633 PMC5771569 29342169

[9] Townzen JS, Brower AVZ, Judd DD. Identification of mosquito blood meals using mitochondrial cytochrome oxidase subunit I and cytochrome b gene sequences. Med Vet Entomol. 2008;22: 386–393. 10.1111/j.1365-2915.2008.00760.x 19120966

[10] Alcaide M, Rico C, Ruiz S, Soriguer R, Muñoz J, Figuerola J. Disentangling vector-borne transmission networks: A universal DNA barcoding method to identify vertebrate hosts from arthropod bloodmeals. PLoS ONE. 2009;4: e7092 10.1371/journal.pone.0007092 19768113 PMC2740869

[11] Townzen JS, Brower AVZ, Judd DD. Identification of mosquito blood meals using mitochondrial *cytochrome oxidase subunit I* and *cytochrome* b gene sequences. Med Vet Entomol. 2008;22: 386–393. 10.1111/j.1365-2915.2008.00760.x 19120966

[12] Ngo KA, Kramer LD. Identification of mosquito blood meals using polymerase chain reaction (PCR) with order-specific primers. J Med Entomol. 2003;40: 215–222. 12693851 10.1603/0022-2585-40.2.215

[13] Cupp EW, Zhang D, Yue X, Cupp MS, Guyer C, Sprenger TR, et al Identification of reptilian and amphibian blood meals from mosquitoes in an *Eastern equine encephalomyelitis virus* focus in central Alabama. Am J Trop Med Hyg. 2004;71: 272–276. 15381805 PMC1351276

[14] Bitome-Essono P-Y, Ollomo B, Arnathau C, Durand P, Mokoudoum ND, Yacka-Mouele L, et al Tracking zoonotic pathogens using blood-sucking flies as 'flying syringes'. eLife. 2017;6: e22069 10.7554/eLife.22069 28347401 PMC5426900

[15] Deagle BE, Jarman SN, Coissac E, Pompanon F, Taberlet P. DNA metabarcoding and the *cytochrome* c *oxidase subunit I* marker: Not a perfect match. Biol Lett. 2014;10: 20140562 10.1098/rsbl.2014.0562 25209199 PMC4190964

[16] Iserte JA, Stephan BI, Goñi SE, Borio CS, Ghiringhelli PD, Lozano ME. Family-specific degenerate primer design: A tool to design consensus degenerated oligonucleotides. Biotechnol Res Int. 2013;2013: 383646 10.1155/2013/383646 23533783 PMC3600133

[17] Compton T. Degenerate primers for DNA amplification In: Innis MA, Gelfand DH, Sninsky JJ, White TJ, editors. PCR Protocols: A guide to methods and applications. San Diego, CA: Academic Press 1990 pp. 39–45.

[18] Linhart C, Shamir R. Degenerate primer design: Theoretical analysis and the HYDEN program In: Walker JM, editor. PCR primer design, methods in molecular biology. Totowa, NJ: Humana Press 2007 pp. 220–244. 10.1007/978-1-59745-528-2_11 17951798

[19] Folmer O, Black M, Hoeh W, Lutz R, Vrijenhoek R. DNA primers for amplification of mitochondrial *cytochrome* c *oxidase subunit I* from diverse metazoan invertebrates Mol Mar Biol Biotechnol. 1994;3: 294–299. 7881515

[20] Clark K, Karsch-Mizrachi I, Lipman DJ, Ostell J, Sayers EW. GenBank. Nucleic Acids Res. 2016;44: D67–D72. 10.1093/nar/gkv1276 26590407 PMC4702903

[21] Kearse M, Moir R, Wilson A, Stones-Havas S, Cheung M, Sturrock S, et al Geneious Basic: An integrated and extendable desktop software platform for the organization and analysis of sequence data. Bioinformatics. 2012;28: 1647–1649. 10.1093/bioinformatics/bts199 22543367 PMC3371832

[22] Nomenclature Committee of the International Union of Biochemistry (NC-IUB). Nomenclature for incompletely specified bases in nucleic acid sequences: Recommendations 1984. Proc Nat Acad Sci. 1986;83: 4–8. 2417239 10.1073/pnas.83.1.4 PMC322779

[23] Kwok S, Kellogg DE, McKinney N, Spasic D, Goda L, Levenson C, et al Effects of primer-template mismatches on the polymerase chain reaction: *Human immunodeficiency virus type 1* model studies. Nucleic Acids Res. 1990;18: 999–1005. 2179874 10.1093/nar/18.4.999 PMC330356

[24] Nagy ZT, Sonet G, Glaw F, Vences M. First large-scale DNA barcoding assessment of reptiles in the biodiversity hotspot of Madagascar, based on newly designed *COI* primers. PLoS ONE. 2012;7: e34506 10.1371/journal.pone.0034506 22479636 PMC3316696

[25] R Development Core Team. R: A language and environment for statistical computing Vienna, Austria: The R Foundation for Statistical Computing 2011.

[26] de Mendiburu F. 2017. Agricolae: Statistical procedures for agricultural research. R package version 1.0–9. 2017. Available from: http://CRAN.R-project.org/package=agricolae

[27] Ratnasingham S, Hebert PDN. A DNA-based registry for all animal species: The Barcode Index Number (BIN) system. PLoS ONE. 2013;8: e66213 10.1371/journal.pone.0066213 23861743 PMC3704603

[28] Hebert PDN, Ratnasingham S, deWaard JR. Barcoding animal life: *Cytochrome* c *oxidase subunit I* divergences among closely related species. Proc R Soc Lond B Biol Sci. 2003;270: S96–S99. 10.1098/rsbl.2003.0025 PMC1698023 12952648

[29] Tollis M, Ausubel G, Ghimire D, Boissinot S. Multi-locus phylogeographic and population genetic analysis of *Anolis carolinensis*: Historical demography of a genomic model species. PLoS ONE. 2012;7: e38474 10.1371/journal.pone.0038474 22685573 PMC3369884

[30] Tollis M, Boissinot S. Genetic variation in the green anole lizard (*Anolis carolinensis*) reveals island refugia and a fragmented Florida during the quaternary. Genetica. 2014;142: 59–72. 10.1007/s10709-013-9754-1 24379168 PMC4778398

[31] Burkett-Cadena ND. A wire-frame shelter for collecting resting mosquitoes. J Am Mosq Control Assoc. 2011;27:153–155. 10.2987/10-6076.1 21805849

[32] Darsie RF, Ward RA. Identification and geographical distribution of the mosquitoes of North America, North of Mexico Gainesville, Florida: The University Press of Florida 2005.

[33] Reeves LE, Holderman CJ, Gillett-Kaufman JL, Kawahara AY, Kaufman PE. Maintenance of host DNA integrity in field-preserved mosquito (Diptera: Culicidae) blood meals for identification by DNA barcoding. Parasit Vectors. 2016;9: 503 10.1186/s13071-016-1791-z 27629021 PMC5024527

[34] Detinova TS. Age-grouping methods in Diptera of medical importance: With special reference to some vectors of malaria. Bull World Health Organ. 1962;47: 48–68. 13885800

[35] Truett GE, Heeger P, Mynatt RL, Truett AA, Walker JA, Warman ML. Preparation of PCR quality mouse genomic DNA with sodium hydroxide and Tris (HotSHOT). BioTechniques. 2000;29: 52–54. 10907076 10.2144/00291bm09

[36] Krysko KL, Burgess JP, Rochford MR, Gillette CR, Cueva D, Enge KM, et al Verified non-indigenous amphibians and reptiles in Florida from 1863 to 2010: Outlining the invasion process and identifying invasion pathways and stages. Zootaxa. 2011;3028: 1–64.

[37] Kent RJ, Norris DE. Identification of mammalian blood meals in mosquitoes by a multiplexed polymerase chain reaction targeting *cytochrome* b. Am J Trop Med Hyg. 2005;73: 336–343. 16103600 PMC4147110

[38] Chow-Schaffer E, Sina B, Hawley WA, De Benedictis J, Scott TW. Laboratory and field evaluation of polymerase chain reaction-based forensic DNA profiling for use in identification of human blood meal sources of *Aedes aegypti* (Diptera: Culicidae). J Med Entomol. 2000;37: 492–502. 10916289 10.1603/0022-2585-37.4.492

[39] Oshaghi MA, Chavshin AR, Vatandoost H, Yaaghoobi F, Mohtarami F, Noorjah N. Effects of post-ingestion and physical conditions on PCR amplification of host blood meal DNA in mosquitoes. Exp Parasitol. 2006;112: 232–236. 10.1016/j.exppara.2005.11.008 16364301

[40] Martínez-de la Puente J, Mendez M, Ruiz S, Godoy JA, Soriguer RC, Figuerola J. Individual identification of endangered species using mosquito blood meals: A proof-of-concept study in Iberian lynx. Parasitol Res. 2015;114: 1607–1610. 10.1007/s00436-015-4343-0 25656463

[41] Deagle BE, Eveson JP, Jarman SN. Quantification of damage in DNA recovered from highly degraded samples–a case study on DNA in faeces. Fron Zool. 2006;3: 11. 10.1186/1742-9994-3-11 PMC1564134 16911807

[42] Meusnier I, Singer GAC, Landry J-F, Hickey DA, Hebert PDN, Hajibabaei M. A universal DNA mini-barcode for biodiversity analysis. BMC Genomics. 2008;9: 214 10.1186/1471-2164-9-214 18474098 PMC2396642

[43] Song H, Buhay JE, Whiting MF, Crandall KA. Many species in one: DNA barcoding overestimates the number of species when nuclear mitochondrial pseudogenes are coamplified. Proc Nat Acad Sci. 2008;105: 13186–13191. 10.1073/pnas.0803076105 PMC2527351 18757756

[44] Richly E, Leister D. NUMTs in sequenced eukaryotic genomes. Mol Biol Evol. 2004;21: 1081–1084. 10.1093/molbev/msh110 15014143

[45] Faraji A, Egizi A, Fonseca DM, Unlu I, Crepeau T, Healy SP, et al Comparative host feeding patterns of the Asian tiger mosquito, *Aedes albopictus*, in urban and suburban northeastern USA and implications for disease transmission. PLoS Negl Trop Dis. 2014;8: e3037 10.1371/journal.pntd.0003037 25101969 PMC4125227

[46] Lutomiah J, Omondi D, Masinga D, Mutai C, Mireji PO, Juliette O, et al Blood meal analysis and virus detection in blood-fed mosquitoes collected during the 2006–2007 Rift Valley fever outbreak in Kenya. Vector Borne Zoonotic Dis. 2014;14: 656–664. 10.1089/vbz.2013.1564 25229704 PMC4171391

[47] Toma T, Miyagi I, Tamashiro M. Blood meal identification and feeding habits of *Uranotaenia* species collected in the Ryukyu Archipelago. J Am Mosquito Contr. 2014;30: 215–218. 10.2987/14-6398R.1 25843097

[48] Brugman VA, Hernández-Triana LM, England ME, Medlock JM, Mertens PPC, Logan JG, et al Blood-feeding patterns of native mosquitoes and insights into their potential role as pathogen vectors in the Thames Estuary region of the United Kingdom. Parasit Vector. 2017;10: 163. 10.1186/s13071-017-2098-4 PMC5369192 28347323

[49] Edman JD. Host-feeding patterns of Florida mosquitoes. I. *Aedes*, *Anopheles*, *Coquillettidia*, *Mansonia* and *Psorophora*. J Med Entomol. 1971;8: 867–895. 10.1093/jmedent/8.6.687 4403447

[50] Edman JD. Host-feeding patterns of Florida mosquitoes: III. *Culex* (*Culex*) and *Culex* (*Neoculex*). J Med Entomol. 1974;11: 95–104. 4828351 10.1093/jmedent/11.1.95

[51] Cupp EW, Zhang D, Yue X, Cupp MS, Guyer C, Sprenger TR, et al Identification of reptilian and amphibian blood meals from mosquitoes in an *Eastern equine encephalomyelitis virus* focus in central Alabama. Am J Trop Med Hyg. 2004;71: 272–276. 15381805 PMC1351276

[52] Burkett-Cadena ND, Graham SP, Hassan HK, Guyer C, Eubanks MD, Katholi CR, et al Blood feeding patterns of potential arbovirus vectors of the genus *Culex* targeting ectothermic hosts. Am J Trop Med Hyg. 2008;79: 809–815. 18981528 PMC4138019

[53] Nasci RS, Edman JD. Blood-feeding patterns of *Culiseta melanura* (Diptera: Culicidae) and associated sylvan mosquitoes in southeastern Massachusetts Eastern equine encephalitis enzootic foci. J Med Entomol. 1981;18: 493–500.

[54] Molaei G, Andreadis TG. Identification of avian-and mammalian-derived bloodmeals in *Aedes vexans* and *Culiseta melanura* (Diptera: Culicidae) and its implication for *West Nile virus* transmission in Connecticut, USA. J Med Entomol. 2006;43: 1088–1093. 10.1603/0022-2585(2006)43[1088:IOAAMB]2.0.CO;2 17017250

[55] Molaei G, Thomas MC, Muller T, Medlock J, Shepard JJ, Armstrong PM, et al Dynamics of vector-host interactions in avian communities in four *Eastern equine encephalitis virus* foci in the northeastern US. PLoS Negl Trop Dis. 2016;10: e0004347 10.1371/journal.pntd.0004347 26751704 PMC4713425

[56] Blosser EM, Lord CC, Stenn T, Acevedo C, Hassan HK, Reeves LE, et al Environmental drivers of seasonal patterns of host utilization by *Culiseta melanura* in Florida. J Med Entomol. 2017;54: 1365–1374. 10.1093/jme/tjx140 28874017 PMC5850491

[57] Logue K, Keven JB, Cannon MV, Reimer L, Siba P, Walker ED, Zimmerman PA, Serre D. Unbiased characterization of *Anopheles* mosquito blood meals by targeted high-throughput sequencing. PLoS Negl Trop Dis. 2016;10: e0004512 10.1371/journal.pntd.0004512 26963245 PMC4786206

[58] Yu G, Fadrosh D, Goedert JJ, Ravel J, Goldstein AM. Nested PCR biases in interpreting microbial community structure in *16S rRNA* gene sequence datasets. PLoS ONE. 2015;10: e0132253 10.1371/journal.pone.0132253 26196512 PMC4509648

[59] Reeves LE, Holderman CJ, Blosser EM, Gillett-Kaufman JL, Kawahara AY, Kaufman PE, Burkett-Cadena ND. Identification of *Uranotaenia sapphirina* as a specialist of annelids broadens known mosquito host use patterns. Commun Biol. 2018;1: 92. 10.1038/s42003-018-0096-5 PMC6123777 30271973

