# Supplementary material for: Barcoding blood meals: New vertebrate-specific primer sets for assigning taxonomic identities to host DNA from mosquito blood meals
Source: PLoS Negl Trop Dis. 2018 Aug 30;12(8):e0006767. doi: 10.1371/journal.pntd.0006767 (PMC6135518; doi:10.1371/journal.pntd.0006767)
Supplement: S1 Table — (DOCX) [file pntd.0006767.s001.docx]

**S1 Table. National Center for Biotechnology Information (NCBI) GenBank accession numbers for mosquito and vertebrate sequences that were aligned and used to identify and design potential vertebrate-specific primers.**

| **Species** | **GenBank Accession Number** |
| --- | --- |
| *Culex quinquefasciatus* | GU188856 |
| *Anopheles quadrimaculatus* | L04272 |
| *Aedes aegypti* | MF194022 |
| *Aedes albopictus* | KX809764 |
| *Uranotaenia sapphirina* | GU908130 |
| *Culex nigripalpus* | KP211393 |
| *Culex coronator* | MF040162 |
| *Culex restuans* | GU908095 |
| *Culex erraticus* | KT766429 |
| *Culex pilosus* | KX779851 |
| *Culex territans* | KR688733 |
| *Culiseta melanura* | JX259979 |
| *Homo sapiens* | MG946759 |
| *Canis latrans* | KT448276 |
| *Felis catus* | U20753 |
| *Procyon lotor* | AM711899 |
| *Didelphis virginiana* | Z29573 |
| *Odocoileus virginianus* | HQ332445 |
| *Sciurus carolinensis* | JF457099 |
| *Rattus rattus* | FJ355927 |
| *Sigmodon hispidus* | JQ601045 |
| *Sylvilagus floridanus* | JQ601063 |
| *Gallus gallus* | KX781319 |
| *Cardinalis cardinalis* | JF498837 |
| *Strix varia* | MF431745 |
| *Buteo lineatus* | KR017961 |
| *Thryothorus ludovicianus* | HM033838 |
| *Ardea herodias* | DQ434302 |
| *Alligator mississippiensis* | Y13113 |
| *Crocodylus acutus* | KF273838 |
| *Tupinambis merianae* | JQ627384 |
| *Anolis carolinensis* | EU747728 |
| *Anolis sagrei* | KP100441 |
| *Python bivittatus* | KF293729 |
| *Agkistrodon piscivorous* | EF669477 |
| *Gopherus polyphemus* | HQ329759 |
| *Lithobates catesbeianus* | AB761267 |
| *Lithobates clamitans* | KY587195 |
| *Lithobates sphenocephalus* | KT388408 |
| *Osteopilus septentrionalis* | KF001943 |
| *Gastrophryne carolinensis* | KT388398 |
| *Hyla squirella* | FJ226851 |
| *Hyla cinerea* | FJ226785 |
