# Supplementary material for: Barcoding blood meals: New vertebrate-specific primer sets for assigning taxonomic identities to host DNA from mosquito blood meals
Source: PLoS Negl Trop Dis. 2018 Aug 30;12(8):e0006767. doi: 10.1371/journal.pntd.0006767 (PMC6135518; doi:10.1371/journal.pntd.0006767)
Supplement: S2 Table — Mosquito-only DNA samples used to ensure that mosquito templates were not amplified are listed under Culicidae. (DOCX) [file pntd.0006767.s002.docx]

**S2 Table. Taxonomic details and collection information for known blood meal samples used to test the effectiveness and versatility of newly designed primers.** Mosquito-only DNA samples used to ensure that mosquito templates were not amplified are listed under Culicidae.

| **Host** | **Mosquito Species** | **Blood Meal Score** | **Collection Date** | **Collection Location** |
| --- | --- | --- | --- | --- |
| MAMMALIA |  |  |  |  |
| **Artiodactylia** |  |  |  |  |
| *Odocoileus virginianus* | *An. quadrimaculatus* | BF1 | 5/5/2017 | Columbia Co., Florida |
| *Sus scrofa* | *Cx. quinquefasciatus* | BF1 | 6/12/2017 | Alachua Co., Florida |
| *Bos taurus* | *Cx. nigripalpus* | BF1 | 7/6/2017 | Alachua Co., Florida |
| **Carnivora** |  |  |  |  |
| *Canis familiaris* | *Cx. quinquefasciatus* | BF1 | 3/27/2017 | Alachua Co., Florida |
| *Canis latrans* | *Cx. nigripalpus* | BF1 | 12/13/2016 | Miami-Dade Co., Florida |
| *Felis catus* | *Cx. quinquefasciatus* | BF1 | 6/21/2017 | Alachua Co., Florida |
| *Procyon lotor* | *Cx. quinquefasciatus* | BF1 | 5/15/2017 | Alachua Co., Florida |
| **Cingulata** |  |  |  |  |
| *Dasypus novemcinctus* | *Ae. infirmatus* | BF1 | 6/20/2017 | Alachua Co., Florida |
| **Didelphimorphia** |  |  |  |  |
| *Didelphis virginiana* | *Cx. erraticus* | BF1 | 5/18/2017 | Alachua Co., Florida |
| **Lagomorpha** |  |  |  |  |
| *Sylvilagus floridanus** | *An. crucians* | BF1 | 9/22/2017 | Alachua Co., Florida |
| *Sylvilagus palustris* | *Ps. ferox* | BF1 | 6/12/2017 | Alachua Co., Florida |
| **Primates** |  |  |  |  |
| *Homo sapiens* | *An. punctipennis* | BF1 | 6/8/2016 | Miami-Dade Co., Florida |
| **Rodentia** |  |  |  |  |
| *Podomys floridanus* | *Cx. cedecei* | BF1 | 5/26/2017 | Miami-Dade Co., Florida |
| *Orozomys palustris* | *Cx. cedecei* | BF1 | 2/8/2017 | Miami-Dade Co. Florida |
| *Rattus rattus* | *Cx. cedecei* | BF1 | 2/8/2017 | Miami-Dade Co. Florida |
| *Sciurus carolinensis* | *Cx. quinquefasciatus* | BF1 | 3/27/2017 | Alachua Co., Florida |
| *Sigmodon hispidus* | *Cx. cedecei* | BF1 | 2/18/2016 | Monroe Co., Florida |
| AVES |  |  |  |  |
| **Accipitriformes** |  |  |  |  |
| *Accipiter cooperi* | *Cx. quinquefasciatus* | BF1 | 3/22/2017 | Alachua Co., Florida |
| *Buteo lineatus* | *Cx. quinquefasciatus* | BF1 | 4/21/2017 | Alachua Co., Florida |
| *Ictinia mississippiensis* | *Cx. nigripalpus* | BF1 | 6/20/2017 | Alachua Co., Florida |
| *Cathartes aura* | *Cx. erraticus* | BF2 | 5/13/2016 | Alachua Co., Florida |
| *Coragyps atratus* | *Cx. quinquefasciatus* | BF1 | 4/27/2017 | Alachua Co., Florida |
| *Pandion haliaetus* | *Cx. nigripalpus* | BF1 | 6/21/2017 | Alachua Co., Florida |
| **Anseriformes** |  |  |  |  |
| *Cairina moschata* | *Cx. nigripalpus* | BF1 | 11/30/2016 | Alachua Co., Florida |
| **Caprimulgiformes** |  |  |  |  |
| *Antrostomus vociferus* | *Cx. quinquefasciatus* | BF1 | 2/24/2017 | Alachua Co., Florida |
| **Ciconiiformes** |  |  |  |  |
| *Mycteria americana* | *Cx. erraticus* | BF2 | 6/9/2016 | Miami-Dade Co, Florida |
| **Columbiformes** |  |  |  |  |
| *Zenaida macroura* | *Cx. nigripalpus* | BF1 | 11/30/2016 | Alachua Co., Florida |
| **Cuculiformes** |  |  |  |  |
| *Coccyzus americanus* | *Cx. quinquefasciatus* | BF2 | 4/21/2017 | Alachua Co., Florida |
| **Gruiformes** |  |  |  |  |
| *Rallus elegans* | *Cx. nigripalpus* | BF1 | 6/9/2016 | Miami-Dade Co., Florida |

| **Passeriformes** |  | |  | |  |  |
| --- | --- | --- | --- | --- | --- | --- |
| *Cardinalis cardinalis* | *Cx. quinquefasciatus* | | BF1 | | 1/11/2017 | Alachua Co., Florida |
| *Corvus brachyrhynchos* | *Cx. quinquefasciatus* | | BF1 | | 5/11/2017 | Alachua Co., Florida |
| *Corvus ossifragus* | *Cx. quinquefasciatus* | | BF2 | | 5/4/2017 | Alachua Co., Florida |
| *Cyanocitta cristata* | *Cx. quinquefasciatus* | | BF1 | | 2/1/2017 | Alachua Co., Florida |
| *Melospiza georgiana* | *Cx. quinquefasciatus* | | BF2 | | 1/15/2017 | Alachua Co., Florida |
| *Spizella passerina* | *Cx. quinquefasciatus* | | BF1 | | 3/1/2017 | Alachua Co., Florida |
| *Haemorhous mexicanus* | *Cx. quinquefasciatus* | | BF1 | | 5/18/2017 | Alachua Co., Florida |
| *Tachycineta bicolor* | *Cs. melanura* | | BF1 | | 3/24/2017 | Alachua Co., Florida |
| *Icterus galbula* | *Cx. restuans* | | BF2 | | 2/13/2017 | Alachua Co., Florida |
| *Dumetella carolinensis* | *Cx. quinquefasciatus* | | BF2 | | 2/3/2017 | Alachua Co., Florida |
| *Mimus polyglottos* | *Cx. quinquefasciatus* | | BF1 | | 5/8/2017 | Alachua Co., Florida |
| *Toxostoma rufum* | *Cx. quinquefasciatus* | | BF1 | | 5/15/2017 | Alachua Co., Florida |
| *Poecile carolinensis* | *Cx. quinquefasciatus* | | BF1 | | 5/17/2017 | Alachua Co., Florida |
| *Setophaga americana* | *Cx. quinquefasciatus* | | BF2 | | 5/11/2017 | Alachua Co., Florida |
| *Setophaga coronata* | *Cx. quinquefasciatus* | | BF1 | | 2/3/2017 | Alachua Co., Florida |
| *Setophaga palmarum* | *Cx. quinquefasciatus* | | BF1 | | 3/1/2017 | Alachua Co., Florida |
| *Setophaga pennsylvanica* | *Cx. quinquefasciatus* | | BF1 | | 3/30/2017 | Alachua Co., Florida |
| *Regulus calendula* | *Cs. melanura* | | BF2 | | 4/5/2017 | Alachua Co., Florida |
| *Thryothorus ludovicianus* | *Cx. nigripalpus* | | BF1 | | 10/26/2016 | Alachua Co., Florida |
| *Troglodytes aedon* | *Cx. quinquefasciatus* | | BF1 | | 2/21/2017 | Alachua Co., Florida |
| *Catharus guttatus* | *Cx. quinquefasciatus* | | BF1 | | 2/21/2017 | Alachua Co., Florida |
| *Catharus ustulatus* | *Cx. quinquefasciatus* | | BF1 | | 5/15/2017 | Alachua Co., Florida |
| *Sialia sialis* | *Cx. quinquefasciatus* | | BF2 | | 5/17/2017 | Alachua Co., Florida |
| *Turdus migratorius* | *Cx. quinquefasciatus* | | BF1 | | 12/1/2015 | Alachua Co., Florida |
| *Myiarchus crinitis* | *Cx. quinquefasciatus* | | BF1 | | 5/10/2017 | Alachua Co., Florida |
| *Sayornis phoebe* | *Cx. quinquefasciatus* | | BF1 | | 1/19/2017 | Alachua Co., Florida |
| *Vireo griseus* | *Cx. nigripalpus* | | BF1 | | 7/14/2017 | Alachua Co., Florida |
| **Pelecaniformes** |  | |  | |  |  |
| *Ardea alba* | *Cx. erraticus* | | BF1 | | 5/26/2017 | Miami-Dade Co., Florida |
| *Ardea herodias* | *Cx. nigripalpus* | | BF1 | | 2/9/2017 | Miami-Dade Co., Florida |
| *Botaurus lentiginosus* | *Cx. erraticus* | | BF2 | | 2/20/2016 | Monroe Co., Florida |
| *Butorides virescens* | *Wy. mitchellii* | | BF1 | | 10/13/15 | Miami-Dade Co., Florida |
| *Egretta thula* | *Cx. erraticus* | | BF2 | | 6/9/2016 | Miami-Dade Co., Florida |
| *Egretta tricolor* | *Cx. erraticus* | | BF1 | | 5/26/2017 | Miami-Dade Co., Florida |
| *Ixobrychus exilis* | *Cx. iolambdis* | | BF1 | | 6/9/2016 | Monroe Co., Florida |
| *Nyctanassa violacea* | *Cx. pilosus* | | BF1 | | 2/20/2017 | Monroe Co., Florida |
| *Eudocimus albus* | *Cx. nigripalpus* | | BF1 | | 6/10/2016 | Miami-Dade Co., Florida |
| **Podicipediformes** |  | |  | |  |  |
| *Podilymbus podiceps* | *Cx. erraticus* | | BF1 | | 10/27/2016 | Alachua Co., Florida |
| **Strigiformes** |  | |  | |  |  |
| *Strix varia* | *Cx. erraticus* | | BF1 | | 2/19/2016 | Monroe-Co., Florida |
| **Suliformes** |  | |  | |  |  |
| *Anhinga anhinga* | *Cx. erraticus* | | BF2 | | 6/9/2016 | Miami-Dade Co., Florida |
| REPTILIA |  | |  | |  |  |
| **Crocodilia** |  | |  | |  |  |
| *Alligator mississippiensis* | *Cx. erraticus* | | BF2 | | 5/5/2017 | Alachua Co., Florida |
| *Crocodylus acutus* | *Cx. pilosus* | | BF1 | | 4/30/2016 | Monroe Co., Florida |
| **Squamata** |  | |  | |  |  |
| *Coluber constrictor* | *Cx. quinquefasciatus* | | BF1 | | 5/18/2017 | Alachua Co., Florida |
| *Nerodia fasciata* | *Cx. iolambdis* | | BF2 | | 6/10/2017 | Miami-Dade Co., Florida |
| *Pantherophis guttatus* | | *Cx. erraticus* | | BF2 | 6/10/2017 | Miami-Dade Co., Florida |
| *Storeria dekayi* | | *Cx. erraticus* | | BF1 | 6/10/2017 | Miami-Dade Co., Florida |
| *Anolis carolinensis* | | *Cx. territans* | | BF1 | 2/13/2017 | Alachua Co., Florida |
| *Anolis equestris* | | *Cx. pilosus* | | BF1 | 12/13/2016 | Miami-Dade Co., Florida |
| *Anolis sagrei* | | *Cx. atratus* | | BF1 | 4/15/2017 | Monroe Co., Florida |
| *Python bivittatus** | | *Cx. erraticus* | | BF1 | 8/17/2016 | Alachua Co., Florida |
| *Salvator merianae** | | *Cx. erraticus* | | BF2 | 8/22/2016 | Alachua Co., Florida |
| *Agkistrodon piscivorus* | | *Cx. erraticus* | | BF1 | 6/9/2016 | Miami-Dade Co., Florida |
| **Testudines** | |  | |  |  |  |
| *Terrapene carolina* | | *Ae. infirmatus* | | BF1 | 6/20/2017 | Alachua Co., Florida |
| *Trachemys scripta* | | *Ae. atlanticus* | | BF1 | 12/1/2015 | Alachua Co., Florida |
| *Gopherus polyphemus* | | *Ae. infirmatus* | | BF1 | 10/28/2016 | Marion Co., Florida |
| *Apalone ferox* | | *Ae. taeniorhynchus* | | BF1 | 5/26/2017 | Miami-Dade Co., Florida |
| AMPHIBIA | |  | |  |  |  |
| **Anura** | |  | |  |  |  |
| *Hyla cinerea* | | *Cx. territans* | | BF1 | 12/28/2016 | Alachua Co., Florida |
| *Hyla squirella* | | *Ur. lowii* | | BF1 | 6/6/2017 | Alachua Co., Florida |
| *Osteopilus septentrionalis* | | *Cx. territans* | | BF1 | 12/29/2016 | Alachua Co., Florida |
| *Pseudacris crucifer* | | *Cx. territans* | | BF2 | 11/26/2016 | Alachua Co., Florida |
| *Gastrophryne carolinensis* | | *Ur. lowii* | | BF1 | 6/6/2016 | Alachua Co., Florida |
| *Lithobates catesbeianus* | | *Cx. territans* | | BF2 | 2/6/2017 | Alachua Co., Florida |
| *Lithobates clamitans* | | *Cx. territans* | | BF1 | 6/23/2017 | Alachua Co., Florida |
| *Lithobates grylio* | | *Ur. lowii* | | BF1 | 5/15/2016 | Indian River Co., Florida |
| *Lithobates sphenocephalus* | | *Ur. lowii* | | BF1 | 5/15/2016 | Indian River Co., Florida |
| CULICIDAE | |  | |  |  |  |
|  | | *Ae. albopictus* | | - | 7/6/2017 | Alachua Co., Florida |
|  | | *Ae. infirmatus* | | - | 7/6/2017 | Alachua Co., Florida |
|  | | *Ae. taeniorhynchus* | | - | 2/9/2017 | Monroe Co. Florida |
|  | | *Ae. triseriatus* | | - | 7/6/2017 | Alachua Co., Florida |
|  | | *An. quadrimaculatus* | | - | 4/30/2016 | Miami-Dade Co., Florida |
|  | | *Cx. coronator* | | - | 7/6/2017 | Alachua Co., Florida |
|  | | *Cx. erraticus* | | - | 7/6/2017 | Alachua Co., Florida |
|  | | *Cx. nigripalpus* | | - | 7/6/2017 | Alachua Co., Florida |
|  | | *Cx. pilosus* | | - | 7/6/2017 | Alachua Co., Florida |
|  | | *Cx. quinquefasciatus* | | - | 9/28/2015 | Alachua Co., Florida |
|  | | *Cx. restuans* | | - | 7/6/2017 | Alachua Co., Florida |
|  | | *Cx. territans* | | - | 4/28/2017 | Alachua Co., Florida |
|  | | *Ur. lowii* | | - | 7/6/2017 | Alachua Co., Florida |
|  | | *Ur. sapphirina* | | - | 7/6/2017 | Alachua Co., Florida |

*Reeves et al. 2018 [8]
