# Supplementary material for: Barcoding blood meals: New vertebrate-specific primer sets for assigning taxonomic identities to host DNA from mosquito blood meals
Source: PLoS Negl Trop Dis. 2018 Aug 30;12(8):e0006767. doi: 10.1371/journal.pntd.0006767 (PMC6135518; doi:10.1371/journal.pntd.0006767)
Supplement: S3 Table — Concentrations were measured by Qubit fluorometer and converted from ng/μl to nM to account for differences in amplicon length between primer combinations. (DOCX) [file pntd.0006767.s003.docx]

**S3 Table. DNA concentration (nM) of PCR products amplified using three primer combinations and templates from various vertebrate host and mosquito species, and negative controls.** Concentrations were measured by QubitTM fluorometer and converted from ng/µl to nM to account for differences in amplicon length between primer combinations.

| **Template category** | **Species** | **Mod_RepCOI_F**  **+**  **Mod_RepCOI_R** | **VertCOI_7194_F**  **+**  **Mod_RepCOI_R** | **Mod_RepCOI_F**  **+**  **VertCOI_7216_R** |
| --- | --- | --- | --- | --- |
| Aves | *Accipiter cooperii* | 185.74 | 181.05 | 369.32 |
| Aves | *Buteo lineatus* | 133.72 | 170.31 | 298.97 |
| Aves | *Ictinia mississippiensis* | 112.27 | 163.41 | 297.62 |
| Aves | *Cathartes aura* | 75.76 | 79.02 | 142.05 |
| Aves | *Coragyps atratus* | 240.96 | 183.35 | 343.61 |
| Aves | *Pandion haliaetus* | 195.78 | 207.13 | 393.67 |
| Aves | *Cairina moschata* | 136.45 | 178.75 | 259.74 |
| Aves | *Antrostomus vociferous* | 196.24 | 164.17 | 362.55 |
| Aves | *Mycteria americana* | 230.92 | 244.73 | 401.79 |
| Aves | *Zenaida macroura* | 162.92 | 169.54 | 236.74 |
| Aves | *Coccyzus americanus* | 169.77 | 214.81 | 367.97 |
| Aves | *Rallus elegans* | 75.76 | 127.35 | 277.33 |
| Aves | *Cardinalis cardinalis* | 156.54 | 157.27 | 303.03 |
| Aves | *Corvus brachyrhynchos* | 163.84 | 147.30 | 238.10 |
| Aves | *Corvus ossifragus* | 133.26 | 144.99 | 215.10 |
| Aves | *Cyanocitta cristata* | 34.55 | 28.54 | 108.09 |
| Aves | *Melospiza georgiana* | 111.35 | 128.12 | 59.79 |
| Aves | *Spizaella passerina* | 229.55 | 197.93 | 263.80 |
| Aves | *Haemorhous mexicanus* | 125.05 | 131.95 | 215.10 |
| Aves | *Tachycineta bicolor* | 106.33 | 175.68 | 263.80 |
| Aves | *Icterus galbula* | 156.99 | 141.16 | 258.39 |
| Aves | *Dumetella carolinensis* | 128.70 | 151.13 | 205.63 |
| Aves | *Mimus polyglottos* | 89.91 | 143.46 | 294.91 |
| Aves | *Toxostoma rufum* | 161.56 | 165.71 | 288.15 |
| Aves | *Poecile carolinensis* | 96.29 | 27.08 | 130.55 |
| Aves | *Setophaga americana* | 152.43 | 193.33 | 232.68 |
| Aves | *Setophaga coronata* | 206.74 | 179.52 | 282.74 |
| Aves | *Setophaga palmarum* | 208.11 | 223.25 | 311.15 |
| Aves | *Setophaga pensylvanica* | 148.78 | 171.85 | 197.51 |
| Aves | *Regulus calendula* | 222.25 | 179.52 | 231.33 |
| Aves | *Thryothorus ludovicianus* | 93.10 | 107.40 | 346.32 |
| Aves | *Troglodytes aedon* | 183.46 | 184.12 | 328.73 |
| Aves | *Catharus guttatus* | 291.62 | 290.76 | 602.00 |
| Aves | *Catharus ustulatus* | 41.30 | 69.97 | 209.69 |
| Aves | *Sialia sialis* | 128.70 | 105.87 | 248.92 |
| Aves | *Turdus migratorius* | 23.41 | 31.07 | 130.55 |
| Aves | *Myiarchus crinitus* | 186.66 | 181.82 | 355.79 |
| Aves | *Sayornis phoebe* | 102.23 | 217.11 | 160.98 |
| Aves | *Vireo griseus* | 141.47 | 115.84 | 396.37 |
| Aves | *Ardea alba* | 193.50 | 232.45 | 346.32 |
| Aves | *Ardea herodias* | 155.62 | 159.57 | 330.09 |
| Aves | *Botaurus lentiginosus* | 114.09 | 171.85 | 265.15 |
| Aves | *Butorides virescens* | 90.36 | 187.96 | 247.56 |
| Aves | *Egretta thula* | 47.69 | 63.37 | 117.56 |
| Aves | *Egretta tricolor* | 179.81 | 171.85 | 338.20 |
| Aves | *Ixobrychus exilis* | 145.58 | 198.70 | 278.68 |
| Aves | *Nyctanassa violacea* | 194.87 | 216.34 | 343.61 |
| Aves | *Eudocimus albus* | 46.09 | 92.83 | 292.21 |
| Aves | *Podilymbus podiceps* | 131.89 | 231.68 | 315.21 |
| Aves | *Strix varia* | 115.46 | 98.96 | 247.56 |
| Aves | *Anhinga anhinga* | 169.31 | 227.85 | 378.79 |
| Amphibia | *Hyla cinereal* | 64.35 | 209.44 | 151.52 |
| Amphibia | *Hyla squirella* | 65.72 | 198.70 | 158.28 |
| Amphibia | *Osteopilus septentrionalis* | 118.20 | 148.06 | 273.27 |
| Amphibia | *Pseudacris crucifer* | 112.72 | 151.90 | 212.39 |
| Amphibia | *Gastrophryne carolinensis* | 183.00 | 151.13 | 178.57 |
| Amphibia | *Lithobates catesbeianus* | 45.64 | 92.06 | 148.81 |
| Amphibia | *Lithobates clamitans* | 64.80 | 127.35 | 103.49 |
| Amphibia | *Lithobates grylio* | 14.97 | 60.68 | 58.31 |
| Amphibia | *Lithobates sphenocephalus* | 71.65 | 126.58 | 75.76 |
| Mammalia | *Bos taurus* | 101.31 | 150.36 | 112.28 |
| Mammalia | *Odocoileus virginianus* | 42.21 | 89.76 | 131.22 |
| Mammalia | *Sus scrofa* | 106.33 | 74.49 | 186.69 |
| Mammalia | *Canis familiaris* | 58.87 | 102.03 | 77.79 |
| Mammalia | *Canis latrans* | 74.39 | 79.79 | 83.87 |
| Mammalia | *Felis catus* | 104.05 | 206.37 | 220.51 |
| Mammalia | *Procyon lotor* | 31.72 | 64.44 | 62.91 |
| Mammalia | *Dasypus novemcinctus* | 164.75 | 169.54 | 196.16 |
| Mammalia | *Didelphis virginiana* | 157.45 | 161.87 | 196.16 |
| Mammalia | *Sylvilagus floridanus* | 97.21 | 137.32 | 132.98 |
| Mammalia | *Sylvilagus palustris* | 37.65 | 95.90 | 64.26 |
| Mammalia | *Homo sapiens* | 163.38 | 153.43 | 235.39 |
| Mammalia | *Podomys floridanus* | 121.85 | 83.62 | 154.22 |
| Mammalia | *Orozomys palustris* | 67.54 | 194.09 | 75.76 |
| Mammalia | *Rattus rattus* | 168.40 | 107.40 | 238.10 |
| Mammalia | *Sciurus carolinensis* | 49.29 | 33.76 | 71.02 |
| Mammalia | *Sigmodon hispidus* | 68.00 | 125.05 | 159.63 |
| Reptilia | *Alligator mississippiensis* | 229.10 | 153.43 | 224.57 |
| Reptilia | *Crocodylus acutus* | 208.56 | 140.39 | 280.03 |
| Reptilia | *Coluber constrictor* | 139.19 | 178.75 | 127.84 |
| Reptilia | *Nerodia fasciata* | 70.74 | 77.48 | 89.96 |
| Reptilia | *Pantherophis guttatus* | 180.27 | 144.23 | 240.80 |
| Reptilia | *Storeria dekayi* | 92.19 | 151.13 | 126.49 |
| Reptilia | *Anolis carolinensis* | 101.31 | 151.13 | 147.46 |
| Reptilia | *Anolis equestris* | 91.73 | 118.91 | 137.99 |
| Reptilia | *Anolis sagrei* | 42.67 | 141.16 | 94.70 |
| Reptilia | *Python bivittatus* | 186.20 | 126.58 | 196.16 |
| Reptilia | *Salvator merianae* | 73.93 | 181.05 | 106.60 |
| Reptilia | *Agkistrodon piscivorus* | 149.69 | 67.13 | 175.87 |
| Reptilia | *Terrapene carolina* | 175.25 | 163.41 | 280.03 |
| Reptilia | *Trachemys scripta* | 43.81 | 58.30 | 116.34 |
| Reptilia | *Gopherus polyphemus* | 168.86 | 174.91 | 239.45 |
| Reptilia | *Apalone ferox* | 86.25 | 186.42 | 126.49 |
| Mosquito | *Aedes albopictus* | 32.86 | 37.74 | 65.21 |
| Mosquito | *Aedes infirmatus* | 26.24 | 35.90 | 21.24 |
| Mosquito | *Aedes taeniorhynchus* | 28.75 | 36.21 | 41.55 |
| Mosquito | *Aedes triseriatus* | 47.46 | 66.67 | 125.27 |
| Mosquito | *Anopheles quadrimaculatus* | 31.17 | 28.08 | 25.03 |
| Mosquito | *Culex coronator* | 31.40 | 36.75 | 41.81 |
| Mosquito | *Culex erraticus* | 22.54 | 36.98 | 44.21 |
| Mosquito | *Culex nigripalpus* | 27.70 | 37.74 | 43.18 |
| Mosquito | *Culex pilosus* | 10.59 | 35.60 | 44.12 |
| Mosquito | *Culex quinquefasciatus* | 22.91 | 25.70 | 38.01 |
| Mosquito | *Culex restuans* | 16.02 | 36.21 | 39.25 |
| Mosquito | *Culex territans* | 22.13 | 38.82 | 27.06 |
| Mosquito | *Uranotaenia lowii* | 17.21 | 45.11 | 88.61 |
| Mosquito | *Uranotaenia sapphirina* | 24.83 | 64.90 | 118.51 |
| Negative | No DNA | 4.88 | 40.42 | 34.09 |
| Negative | No DNA | 7.12 | 40.12 | 35.33 |
| Negative | No DNA | 8.31 | 35.83 | 43.97 |
| Negative | No DNA | 7.32 | 38.28 | 35.98 |
