# Supplementary material for: Barcoding blood meals: New vertebrate-specific primer sets for assigning taxonomic identities to host DNA from mosquito blood meals
Source: PLoS Negl Trop Dis. 2018 Aug 30;12(8):e0006767. doi: 10.1371/journal.pntd.0006767 (PMC6135518; doi:10.1371/journal.pntd.0006767)
Supplement: S4 Table — (DOCX) [file pntd.0006767.s004.docx]

**S4 Table. Mosquitoes collected at River Styx, Alachua Co., FL, USA, digestion extent and vertebrate host identification.**

| ID | Mosquito sp | Digestion extent | Host sp. |
| --- | --- | --- | --- |
| RS45 | *Aedes infirmatus* | BF1 | *Odocoileus virginianus* |
| RS46 | *Aedes infirmatus* | BF1 | *Odocoileus virginianus* |
| RS53 | *Aedes infirmatus* | BF1 | *Odocoileus virginianus* |
| RS54 | *Aedes infirmatus* | BF1 | *Odocoileus virginianus* |
| RS55 | *Aedes infirmatus* | BF1 | *Odocoileus virginianus* |
| RS56 | *Aedes infirmatus* | BF2 | *Odocoileus virginianus* |
| RS57 | *Aedes infirmatus* | BF1 | *Odocoileus virginianus* |
| RS58 | *Aedes infirmatus* | BF3 | *Odocoileus virginianus* |
| RS59 | *Aedes infirmatus* | BF3 | *Odocoileus virginianus* |
| RS60 | *Aedes infirmatus* | BF1 | *Odocoileus virginianus* |
| RS07 | *Aedes infirmatus* | BF2 | *Dasypus novemcinctus* |
| RS61 | *Aedes infirmatus* | BF3 | *Dasypus novemcinctus* |
| RS50 | *Aedes infirmatus* | BF3 | *Sylvilagus palustris* |
| RS62 | *Aedes infirmatus* | BF1 | *Sylvilagus palustris* |
| RS26 | *Anopheles crucians* | BF1 | *Odocoileus virginianus* |
| RS35 | *Coquillettiddia perturbans* | BF3 | *Odocoileus virginianus* |
| RS42 | *Coquillettiddia perturbans* | BF2 | *Odocoileus virginianus* |
| RS43 | *Coquillettiddia perturbans* | BF3 | *Odocoileus virginianus* |
| RS44 | *Coquillettiddia perturbans* | BF1 | *Odocoileus virginianus* |
| RS04 | *Coquillettiddia perturbans* | BF3 |  |
| RS01 | *Culex erraticus* | BF2 | *Butorides virescens* |
| RS08 | *Culex erraticus* | BF1 | *Nycticorax nycticorax* |
| RS41 | *Culex erraticus* | BF1 | *Nycticorax nycticorax* |
| RS02 | *Culex territans* | BF1 | *Hyla cinerea* |
| RS16 | *Culex territans* | BF1 | *Hyla cinerea* |
| RS20 | *Culex territans* | BF1 | *Hyla cinerea* |
| RS22 | *Culex territans* | BF2 | *Hyla cinerea* |
| RS24 | *Culex territans* | BF3 | *Hyla cinerea* |
| RS29 | *Culex territans* | BF1 | *Hyla cinerea* |
| RS03 | *Culex territans* | BF1 | *Hyla femoralis* |
| RS05 | *Culex territans* | BF2 | *Hyla femoralis* |
| RS40 | *Culex territans* | BF2 | *Hyla femoralis* |
| RS49 | *Culex territans* | BF1 | *Hyla squirella* |
| RS06 | *Culex territans* | BF1 | *Anolis carolinensis* |
| RS12 | *Culex territans* | BF1 | *Anolis carolinensis* |
| RS13 | *Culex territans* | BF1 | *Anolis carolinensis* |
| RS14 | *Culex territans* | BF1 | *Anolis carolinensis* |
| RS15 | *Culex territans* | BF1 | *Anolis carolinensis* |
| RS17 | *Culex territans* | BF1 | *Anolis carolinensis* |
| RS18 | *Culex territans* | BF1 | *Anolis carolinensis* |
| RS19 | *Culex territans* | BF1 | *Anolis carolinensis* |
| RS21 | *Culex territans* | BF1 | *Anolis carolinensis* |
| RS23 | *Culex territans* | BF2 | *Anolis carolinensis* |
| RS27 | *Culex territans* | BF1 | *Anolis carolinensis* |
| RS28 | *Culex territans* | BF1 | *Anolis carolinensis* |
| RS30 | *Culex territans* | BF1 | *Anolis carolinensis* |
| RS31 | *Culex territans* | BF2 | *Anolis carolinensis* |
| RS32 | *Culex territans* | BF2 | *Anolis carolinensis* |
| RS33 | *Culex territans* | BF2 | *Anolis carolinensis* |
| RS37 | *Culex territans* | BF2 | *Anolis carolinensis* |
| RS38 | *Culex territans* | BF2 | *Anolis carolinensis* |
| RS39 | *Culex territans* | BF2 | *Anolis carolinensis* |
| RS47 | *Culex territans* | BF1 | *Anolis carolinensis* |
| RS48 | *Culex territans* | BF2 | *Anolis carolinensis* |
| RS34 | *Culex territans* | BF2 | *Anolis carolinensis* |
| RS25 | *Culex territans* | BF0 |  |
| RS63 | *Culex territans* | BF0 |  |
| RS09 | *Culiseta melanura* | BF2 | *Cardinalis cardinalis* |
| RS10 | *Culiseta melanura* | BF1 | *Anolis carolinensis* |
| RS11 | *Culiseta melanura* | BF1 | *Anolis carolinensis* |
| RS36 | *Culiseta melanura* | BF1 | *Anolis carolinensis* |
| RS51 | *Culiseta melanura* | BF3 | *Anolis carolinensis* |
| RS52 | *Culiseta melanura* | BF1 | *Anolis carolinensis* |
| RS45 | *Aedes infirmatus* | BF1 | *Odocoileus virginianus* |
